# MiR-124 sensitizes cisplatin-induced cytotoxicity against CD133^+^ hepatocellular carcinoma cells by targeting SIRT1/ROS/JNK pathway

**DOI:** 10.18632/aging.101876

**Published:** 2019-05-05

**Authors:** Yunxiuxiu Xu, Yu Lai, Hanqin Weng, Lanping Tan, Yanshan Li, Guangcheng Chen, Xingxi Luo, Yibiao Ye

**Affiliations:** 1 Department of Hepato-Billiary Surgery, Sun Yat-sen Memorial Hospital, Sun Yat-sen University, Guangzhou 510120, China; 2 Department of Gastroenterology, Sun Yat-sen Memorial Hospital, Sun Yat-sen University, Guangzhou 510120, China; 3 Department of Hepato-Billiary Surgery, Dongguan people’s Hospital, Southern Medical University, Guangdong 523905, China; 4 Department of Thyroid Surgery, Sun Yat-sen Memorial Hospital, Sun Yat-sen University, Guangzhou 510120, China; 5 Department of Blood Transfusion, Sun Yat-sen Memorial Hospital, Sun Yat-sen University, Guangzhou 510120, China; 6 Department of Gastrointestinal Surgery, Sun Yat-sen Memorial Hospital, Sun Yat-sen University, Guangzhou 510120, China; 7 Key Laboratory of Malignant Tumor Gene Regulation and Target Therapy of Guangdong Higher Education Institutes, Sun Yat-sen Memorial Hospital, Sun Yat-sen University, Guangzhou 510120, China; * Equal contribution

**Keywords:** miR-124, SIRT1, ROS, JNK, cisplatin, HCC

## Abstract

Drug resistance is still a major obstacle for efficient treatment of hepatocellular carcinoma (HCC) during the cisplatin-based chemotherapy. Recent studies have demonstrated that CD133 positive population of cancer cells are responsible for multiple drug resistance. We are supposed to take strategies to sensitize CD133^+^ HCC cells to cisplatin treatment. In the present study, CD133^+^ HCC cells showed significant cisplatin-resistance compared to the CD133^-^ HCC cells. Downregulation of miR-124 was observed in CD133^+^ HCC cells. However, enforced expression of miR-124 can increase the sensitivity of CD133^+^ HCC cells to cisplatin treatment in vitro and in vivo. Mechanically, overexpression of miR-124 was found to inhibit the expression of SIRT1 and thus promoted the generation of ROS and phosphorylation of JNK. As the results, overexpression of miR-124 expanded the apoptosis in cisplatin-treated CD133^+^ HCC cells. We then demonstrated that overexpression of miR-124 sensitized cisplatin-induced cytotoxicity against CD133^+^ hepatocellular carcinoma cells by targeting SIRT1/ROS/JNK pathway.

## Introduction

Hepatocellular carcinoma (HCC) is one of the most commonly diagnosed malignancy and the leading cause of cancer-related death in the world [1,2]. Despite advances of modern treatments, chemotherapy is still a main and effective approach to control the development of HCC and prolong patients’ life. However, many HCC cases show poor response to chemotherapy [3,4]. Chemoresistance is still a major obstacle for HCC patients to obtain a satisfactory curative effect. Recently, studies demonstrate that a population of CD133^+^ cancer cells exhibited significant resistance against chemotherapy in some cancers [5–7].

In human cells, CD133 is a pentaspan transmembrane glycoprotein encoded by the prominin 1 (PROM1) gene. In some cancers, CD133 has been identified as an important molecular marker of stem-like cells and called cancer stem cells (CSCs). Studies have reported that CD133^+^ cancer cells display high tumorigenicity, proliferation, self-renewal and drug resistance [8–11]. CD133^+^ cancer cells have become important targets in cancer therapy.

MicroRNAs (miRNAs) are a class of endogenously expressed and non-coding small RNAs. Cellular miRNAs act as gene suppressors through binding to 3’untranslated region (3’ UTR) of targeted mRNAs followed by induction of translational repression [12–14]. As approximate 60% of all human genes were regulated by miRNAs, miRNAs participate in various biological processes including cell proliferation, differentiation, migration, self-renewal, metabolism and apoptosis in cancer cells [15,16]. Furthermore, studies have demonstrated that miRNA dysregulation is a frequent incidence and is responsible for chemoresistance in various cancers including HCC [17–19]. Correction of miRNA disorder may be a potential approach for improving the cancer therapy.

SIRT1 is a histone deacetylase in human cells. As SIRT1 has been reported to act as a potential oncogene, it is usually overexpressed in multiple human cancers including hepatocellular carcinoma, osteosarcoma, lung cancer and colorectal cancer [20–24]. Furthermore, Overexpression of SIRT1 is found to promote cancer development and implicate poor prognosis in cancer patients [25,26]. Recently, studies also demonstrate that SIRT1 overexpression is responsible for chemoresistance in cancers [27,28]. SIRT1 has become a potential target in cancer treatment. In the present study, we found that SIRT1 was overexpressed and was targeted by miR-124 in CD133^+^ HCC cells. The aim of this study is to explore the effect of miR-124/SIRT1 axis on cisplatin-based chemotherapy against CD133^+^ HCC cells.

## RESULTS

### CD133^+^ HCC cells exhibit resistance to cisplatin

**Figure 1:**
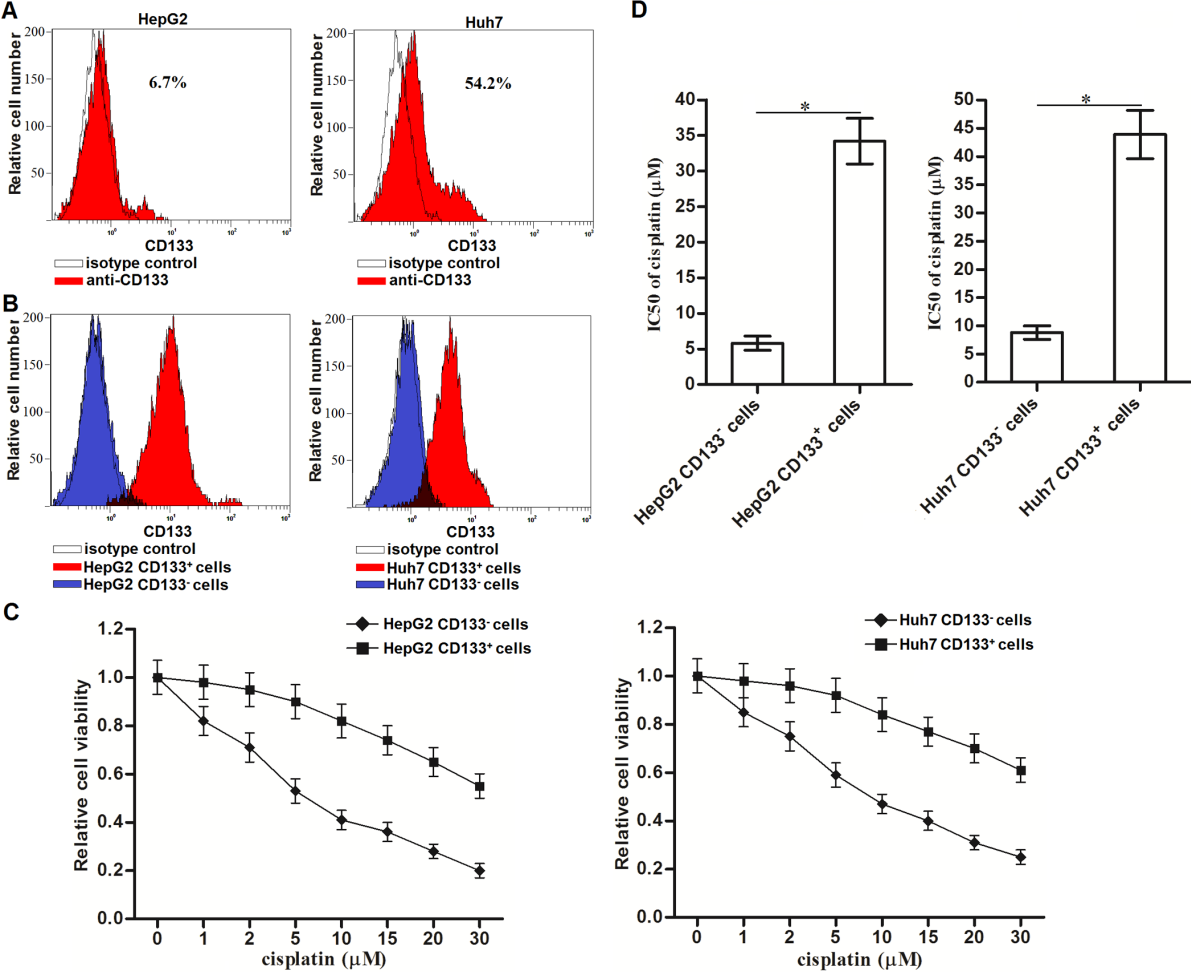
**Cisplatin resistance of CD133^+^ HCC cells.** (**A**) CD133^+^ HCC cells were identified by using CD133 antibody in HepG2 and Huh7 cells. (**B**) Purity of sorted CD133^+^ and CD133^-^ HepG2 and Huh7 cells was tested by flow cytometry. (**C**) CD133^+^ and CD133^-^ HepG2 and Huh7 cells were treated with different concentrations of cisplatin (0~30 μM) for 48 h. Cell viability curve was conducted by MTT assays. (**D**) IC50 of cisplatin to CD133^+^ and CD133^-^ HepG2 and Huh7 cells was calculated according to the MTT assays. *P<0.05.

To investigate the drug resistance of CD133 positive HCC cells, we first sorted and collected the CD133^+^ and CD133^-^ population of HepG2 and Huh7 cells through flow cytometry (Figure 1A). The purity of CD133^+^ and CD133^-^ cells was tested by using the CD133-FITC antibody (Figure 1B). Results of MTT assays showed that the sensitivity of CD133^+^ HepG2 and Huh7 cells to cisplatin was obviously lower than the CD133^-^ HCC cells when they were under the equal concentrations of cisplatin (Figure 1C). Specifically, IC50 of cisplatin to CD133^+^ HepG2 cells was 5.02 fold higher than the IC50 of cisplatin to CD133^-^ HepG2 cells. Meanwhile, IC50 of cisplatin to CD133^+^ Huh7 cells was 4.09 fold higher than the IC50 of cisplatin to CD133^-^ Huh7 cells (Figure 1D). These results indicated that CD133^+^ HCC cells exhibit resistance to cisplatin.

### Overexpression of SIRT1 is responsible for the cisplatin resistance in CD133^+^ HCC cells

**Figure 2:**
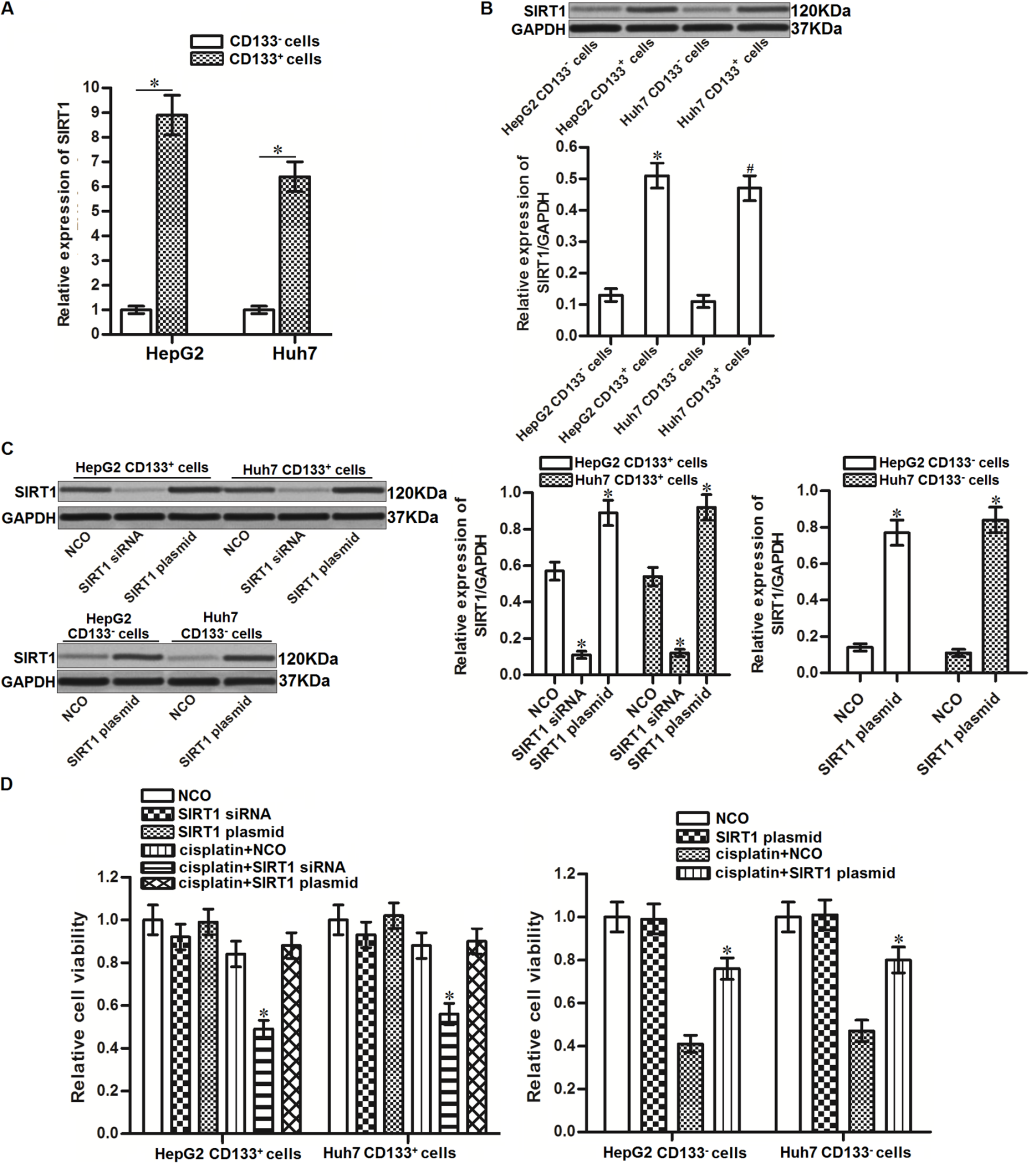
**Effect of SIRT1 on regulating the sensitivity of cisplatin to HCC cells.** (**A**) qRT-PCR analysis was performed to detect the expression of SIRT1 in CD133^+^ and CD133^-^ HepG2 and Huh7 cells at the mRNA level. *P<0.05. (**B**) Western blot analysis was performed to detect the expression of SIRT1 in CD133^+^ and CD133^-^ HepG2 and Huh7 cells at the protein level. *P<0.05 *vs.* HepG2 CD133^-^ cells. ^#^*P*<0.05 *vs.* Huh7 CD133^-^ cells. (**C**) Transfection efficiency of SIRT1 siRNA (50 pmol/mL) and plasmid (2 μg/mL) in CD133^+/-^ HepG2 and Huh7 cells after 24 h transfection. *P<0.05 *vs.* NCO group. (**D**) CD133^+^ and CD133^-^ HepG2 and Huh7 cells were transfected with SIRT1 siRNA (50 pmol/mL) or plasmid (2 μg/mL). 24h later, these cells were treated with cisplatin (10 μM) for another 48 h. MTT assays were performed to measure the cell viability of these cells. *P<0.05 *vs.* cisplatin + NCO group.

Results of qRT-PCR and western blot analysis showed that CD133^+^ HepG2 and Huh7 cells expressed obviously higher level of SIRT1 compared to CD133^-^ HepG2 and Huh7 cells at the mRNA level (Figure 2A) and protein level (Figure 2B), respectively. To investigate whether the overexpression of SIRT1 is responsible for the cisplatin resistance of CD133^+^ HepG2 and Huh7 cells, we performed the loss-of-function and gain-of-function assays in HCC cells. The effect of SIRT1 siRNA and plasmid in CD133^+/-^ HepG2 and Huh7 cells were shown in Figure 2C. Despite CD133^+^ HepG2 and Huh7 cells were not sensitive to SIRT1 overexpression, results of cytotoxicity assays showed that transfection with SIRT1 siRNA increased the sensitivity of CD133^+^ HepG2 and Huh7 cells to cisplatin treatment. On the contrary, transfection with SIRT1 plasmid induced resistance to cisplatin in CD133^-^ HepG2 and Huh7 cells (Figure 2D). These data indicated that overexpression of SIRT1 is responsible for the cisplatin resistance in CD133^+^ HCC cells.

### SIRT1 is targeted by miR-124 in HCC

**Figure 3:**
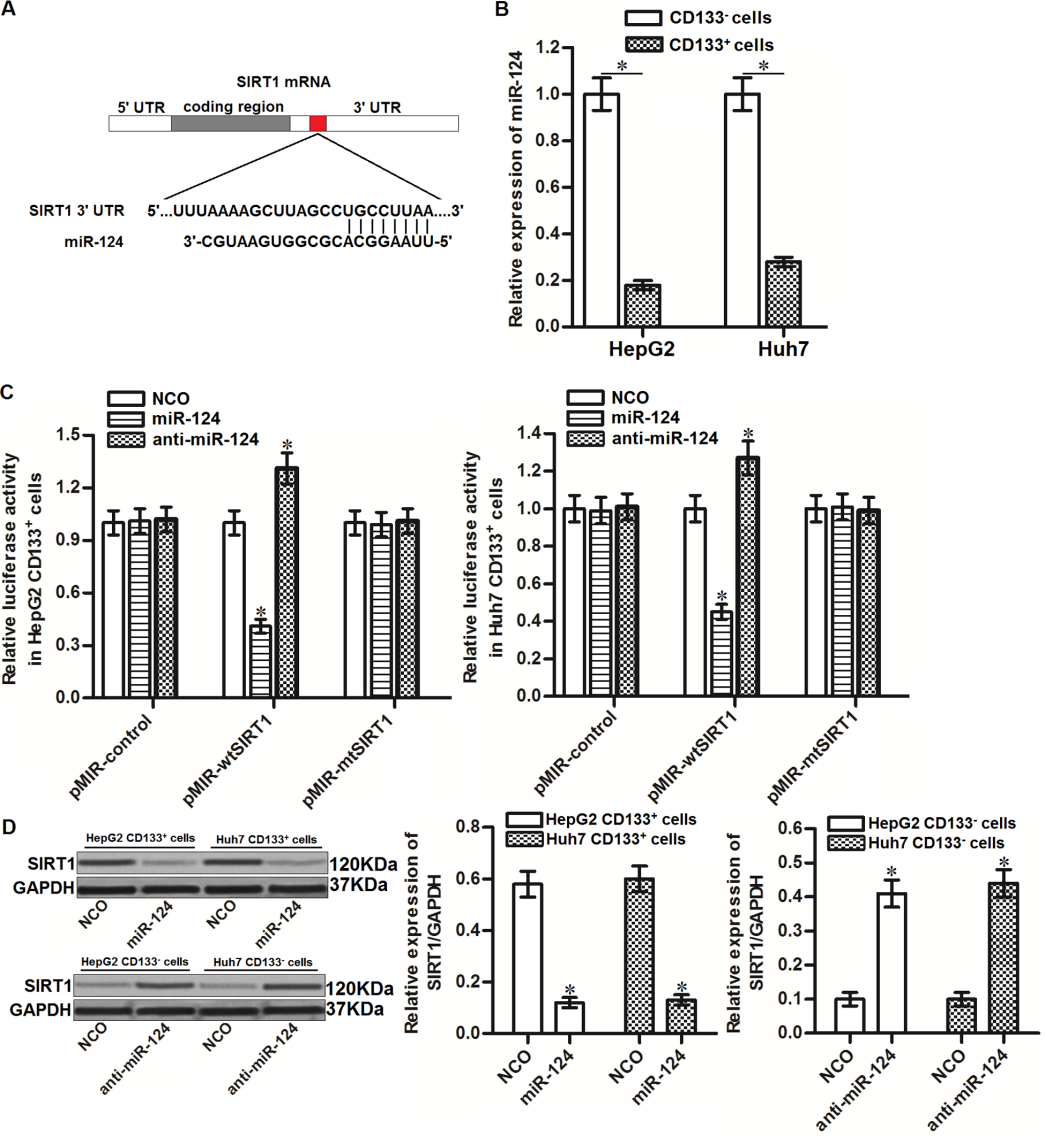
**SIRT1 is targeted by miR-124 in HCC.** (**A**) Complementary sequence of SIRT1 3’ UTR paired with miR-124. (**B**) Expression of miR-124 in CD133^+^ and CD133^-^ HepG2 and Huh7 cells was detected by qRT-PCR. (**C**) Effect of miR-124 mimics and inhibitors on changing the luciferase activities of pMIR plasmid contained SIRT1 3’ UTR. *P<0.05 *vs.* NCO group. (**D**) CD133^+^ and CD133^-^ HepG2 and Huh7 cells were transfected with miR-124 (50 pmol/mL) or anti-miR-124 (50 pmol/mL) for 24h. Western blot analysis was then performed to detect the expression of SIRT1 in these cells. *P<0.05 *vs.* NCO group.

To investigate the mechanism by which SIRT1 was overexpressed in CD133^+^ HCC cells, public miRNA prediction databases of TargetScan, miRanda and PicTar were used to search the upstream miRNA of SIRT1. All of these databases showed that 3’ UTR of SIRT1 gene contained highly conserved sequence paired with miR-124 (Figure 3A). Furthermore, in contrast with upregulation of SIRT1 in CD133^+^ HCC cells, expression of miR-124 was significantly decreased in CD133^+^ HepG2 and Huh7 cells (Figure 3B). We thus predicted that SIRT1 was targeted by miR-124 in HCC. To confirm this speculation, we performed luciferase reporter assays after co-transfection with miR-124 mimics or inhibitors and pMIR plasmid contained SIRT1 3’ UTR. The results showed that co-transfection with miR-124 mimics was able to decrease the luciferase activities of pMIR reporters contained with wild type SIRT1 (pMIR-wtSIRT1) but not the empty reporters or pMIR reporters contained with mutant type SIRT1 (pMIR-mtSIRT1). Besides, co-transfection with anti-miR-124 increased the luciferase activities of pMIR-wtSIRT1 (Figure 3C). Furthermore, results of western blot analysis showed that recovery of miR-124 was found to decrease the protein level of SIRT1 in CD133^+^ HepG2 and Huh7 cells, whereas knockdown of miR-124 in CD133^-^ HepG2 and Huh7 cells increased the protein level of SIRT1 (Figure 3D). Taken together, these results indicated that SIRT1 was targeted by miR-124 in HCC.

### Overexpression of miR-124 sensitizes the CD133^+^ HCC cells to cisplatin through suppression of SIRT1

**Figure 4:**
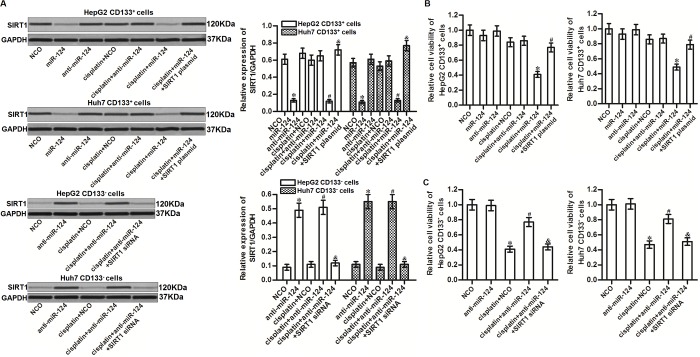
**MiR-124 sensitizes HCC cells to cisplatin through suppression of SIRT1.** (**A**) CD133^+^ and CD133^-^ HepG2 and Huh7 cells were transfected with miR-124 (50 pmol/mL), anti-miR-124 (50 pmol/mL), SIRT1 siRNA (50 pmol/mL) and SIRT1 plasmid (2 μg/mL). 24h later, these cells were treated with cisplatin (10 μM) for another 48 h. Expression of SIRT1 in these cells was then detected by western blot analysis. *P<0.05 *vs.* NCO group. ^#^*P*<0.05 *vs.* cisplatin + NCO group. ^&^*P*<0.05 *vs.* cisplatin + miR-124 group. (**B**) CD133^+^ HepG2 and Huh7 cells were transfected with miR-124 (50 pmol/mL) and SIRT1 plasmid (2 μg/mL). 24h later, these cells were treated with cisplatin (10 μM) for another 48 h. Cell viability was then detected by MTT assays. *P<0.05 *vs.* cisplatin + NCO group. ^#^*P*<0.05 *vs.* cisplatin + miR-124 group. (**C**) CD133^-^ HepG2 and Huh7 cells were transfected with anti-miR-124 (50 pmol/mL) and SIRT1 siRNA (50 pmol/mL). 24h later, these cells were treated with cisplatin (10 μM) for another 48 h. Cell viability was then detected by MTT assays. *P<0.05 *vs.* NCO group. ^#^*P*<0.05 *vs.* cisplatin + NCO group. ^&^*P*<0.05 *vs.* cisplatin + miR-124 group.

To investigate the role of miR-124/SIRT1 axis in changing the sensitivity of CD133^+^ HCC cells to cisplatin, we transfected the CD133^+^ HepG2 and Huh7 cells with miR-124 and SIRT1 plasmid before treatment with cisplatin. Effect of miR-124, SIRT1 plasmid and cisplatin on changing the SIRT1 expression was shown in Figure 4A. Results of MTT assays showed that transfection with miR-124 significantly enhanced the cytotoxicity of cisplatin to CD133^+^ HepG2 and Huh7 cells. However, enforced expression of SIRT1 decreased the effect of miR-124 on sensitizing the CD133^+^ HepG2 and Huh7 cells to cisplatin (Figure 4B). Besides, we found that knockdown of miR-124 by using anti-miR-124 induced resistance of cisplatin in CD133^-^ HepG2 and Huh7 cells (Figure 4C). Taken together, these results indicated that miR-124/SIRT1 axis increased cisplatin sensitivity in HCC cells. Recovery of miR-124 expression was able to sensitize the CD133^+^ HCC cells to cisplatin through suppression of SIRT1.

### Effect of miR-124 on sensitizing the cisplatin-induced cytotoxicity to CD133^+^ HCC was dependent on the ROS pathway

**Figure 5:**
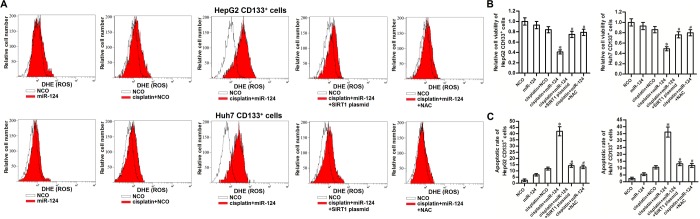
**miR-124 enhanced the cisplatin-induced cytoxocity against CD133^+^ HCC cells through SIRT1/ROS pathway.** (**A**) CD133^+^ HepG2 and Huh7 cells were treated with miR-124 (50 pmol/mL), SIRT1 plasmid (2 μg/mL) and NAC (2 mM). 24h later, these cells were treated with cisplatin (10 μM) for another 48 h. Cellular ROS was detected by flow cytometry. (**B**) CD133^+^ HepG2 and Huh7 cells were treated with miR-124 (50 pmol/mL), SIRT1 plasmid (2 μg/mL) and NAC (2 mM). 24h later, these cells were treated with cisplatin (10 μM) for another 48 h. Cell viability was detected by MTT assays. *P<0.05 *vs.* cisplatin + NCO group. ^#^*P*<0.05 *vs.* cisplatin + miR-124 group. (**C**) CD133^+^ HepG2 and Huh7 cells were treated with miR-124 (50 pmol/mL), SIRT1 plasmid (2 μg/mL) and NAC (2 mM). 24h later, these cells were treated with cisplatin (10 μM) for another 48 h. Flow cytometry analysis was then performed to detect the cell apoptotic rate. *P<0.05 *vs.* cisplatin + NCO group. ^#^*P*<0.05 *vs.* cisplatin + miR-124 group.

SIRT1 has been reported to prevent the generation of ROS in human cells [29]. We thus tested the role of ROS in miR-124-promoted cell death in cisplatin-treated CD133^+^ HCC cells. Results of flow cytometry analysis showed that combination with miR-124 increased the production of ROS in the cisplatin-treated CD133^+^ HepG2 and Huh7 cells. However, overexpression of SIRT1 was found to abolish the effect of miR-124 on promoting the ROS generation (Figure 5A). It indicated that miR-124 was able to promote the ROS production through the SIRT1 pathway. Next, we evaluated the role of ROS in cisplatin-induced cytotoxicity to CD133^+^ HCC by using the N-acetylcysteine (NAC) which is used as a ROS scavenger [30]. Our data showed that NAC significantly improve the cell viability of CD133^+^ HepG2 and Huh7 cells which were co-treated with cisplatin and miR-124 (Figure 5B). Furthermore, despite combination with cisplatin and miR-124 induced significant apoptosis of CD133^+^ HepG2 and Huh7 cells, we showed that either SIRT1 plasmid or NAC can protect these HCC cells from the cytotoxicity of cisplatin and miR-124 co-treatment (Figure 5C). Taken together, we demonstrated that miR-124 increased the sensitivity of CD133^+^ HCC cells to cisplatin-induced apoptosis through the SIRT1/ROS pathway.

### JNK is the downstream of miR-124/SIRT1/ROS pathway in CD133^+^ HCC cells

**Figure 6:**
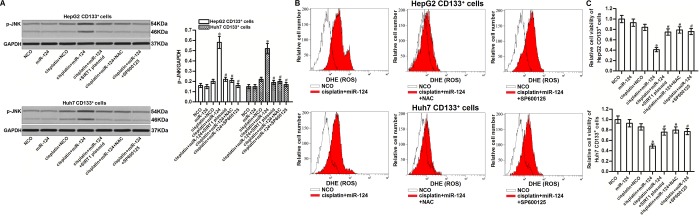
**JNK is the downstream of miR-124/SIRT1/ROS pathway in CD133^+^ HCC cells.** (**A**) CD133^+^ HepG2 and Huh7 cells were treated with miR-124 (50 pmol/mL), SIRT1 plasmid (2 μg/mL), NAC (2 mM) and SP600125 (50 μM). 24h later, these cells were treated with cisplatin (10 μM) for another 48 h. Phosphorylated JNK in these cells was then detected by western blot analysis. *P<0.05 *vs.* cisplatin + NCO group. ^#^*P*<0.05 *vs.* cisplatin + miR-124 group. (**B**) CD133^+^ HepG2 and Huh7 cells were treated with miR-124 (50 pmol/mL), SIRT1 plasmid (2 μg/mL), NAC (2 mM) and SP600125 (50 μM). 24h later, these cells were treated with cisplatin (10 μM) for another 48 h. Cellular ROS was then detected by flow cytometry. (**C**) CD133^+^ HepG2 and Huh7 cells were treated with miR-124 (50 pmol/mL), SIRT1 plasmid (2 μg/mL), NAC (2 mM) and SP600125 (50 μM). 24h later, these cells were treated with cisplatin (10 μM) for another 48 h. Cell viability cells was measured by MTT assays. *P<0.05 *vs.* cisplatin + NCO group. ^#^*P*<0.05 *vs.* cisplatin + miR-124 group.

It has been reported that JNK is a molecular linkage between oxidative stress and cell apoptosis [31]. We thus tested the role of JNK in miR-124-promoted cell death in cisplatin-treated CD133^+^ HCC cells. Results of western blot analysis showed that combination with miR-124 enhanced the phosphorylation of JNK in the cisplatin-treated CD133^+^ HCC cells. However, we found that either SIRT1 plasmid or NAC could inhibit the phosphorylation of JNK (Figure 6A). Furthermore, we found that SP600125 which is used as a inhibitor of JNK [32] failed to prevent the ROS production in the cisplatin and miR-124 co-treated CD133^+^ HepG2 and Huh7 cells (Figure 6B). It indicated that the SIRT1/ROS pathway was the upstream of JNK in CD133^+^ HCC cells. Next, we showed that SP600125 could protect CD133^+^ HepG2 and Huh7 cells from the cytotoxicity of cisplatin and miR-124 co-treatment (Figure 6C). Taken together, we demonstrated that JNK is the downstream of miR-124/SIRT1/ROS pathway. Activation of JNK is essential for the miR-124-promoted cell death in cisplatin-treated CD133^+^ HCC cells.

### Overexpression of miR-124 decreases the resistance of CD133^+^ HCC cells to cisplatin treatment in vivo

**Figure 7:**
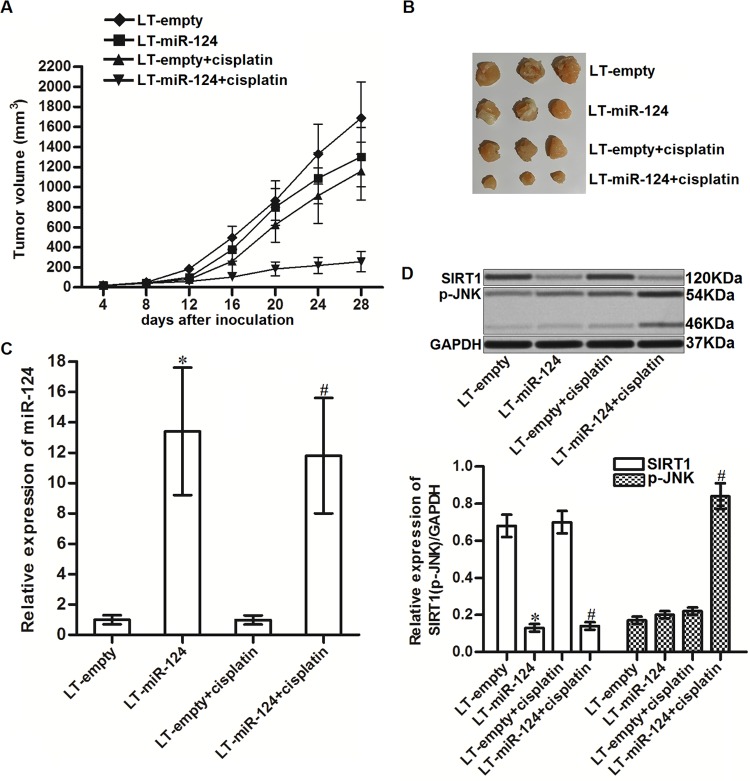
**MiR-124 enhanced the cytotoxicity of cisplatin to CD133^+^ HCC tumors in vivo.** (**A**) Tumor growth of CD133^+^ HepG2 tumors treated with cisplatin (5 mg/kg). (**B**) Separated tumors after 28 days inoculation. (**C**) Expression of miR-124 in resected tumor tissues. *P<0.05 *vs.* LT-empty. ^#^*P*<0.05 *vs.* LT-empty + cisplatin group. (**D**) Expression of SIRT1 and phosphorylated JNK in resected tumor tissues. *P<0.05 *vs.* LT-empty. ^#^*P*<0.05 *vs.* LT-empty + cisplatin group.

To investigate the role of miR-124 in resistance of CD133^+^ HCC cells to cisplatin treatment in vivo, we inoculated the nude mice with the CD133^+^ HepG2 cells transfected with lentivirus carrying miR-124 precursor (LT-miR-124). Under the administration of equal dose of cisplatin (5 mg/kg), the tumor growth of miR-124-overexpressed CD133^+^ HepG2 cells was obviously slower than the control CD133^+^ HepG2 tumors (Figure 7A). Finally, the sizes of cisplatin and miR-124 co-treated tumors were smaller than the control tumors treated with equal dose of cisplatin (Figure 7B). After euthanasia of mice, we found significant upregulation of miR-124 in LT-miR-124-transfected tumors (Figure 7C). In contrast, expression of SIRT1 was decreased in the LT-miR-124-transfected tumors. In addition, we found that miR-124 enhanced the phosphorylation of JNK in the cisplatin-treated CD133^+^ HepG2 tumors (Figure 7D). Taken together, we demonstrated that overexpression of miR-124 could decrease the resistance of CD133^+^ HCC cells to cisplatin treatment in vivo.

### Overexpression of miR-124 decreases the resistance of CD133^+^ HCC cells to other platinum-based chemotherapeutic drugs

**Figure 8:**
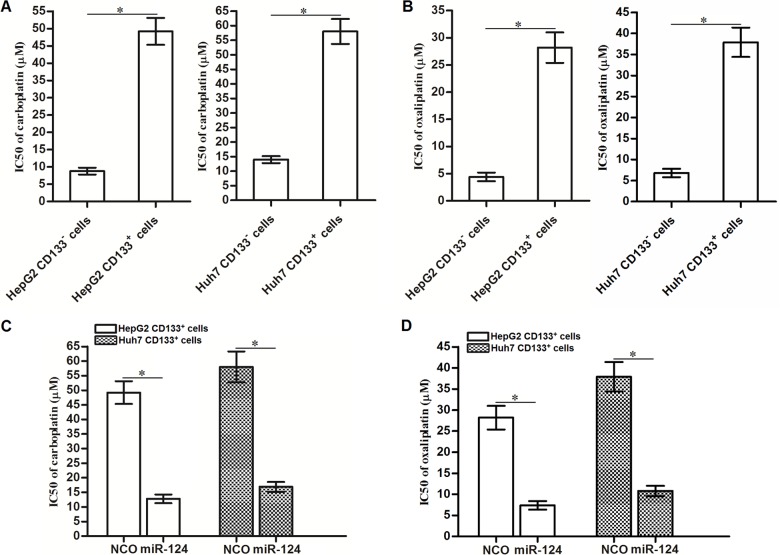
**Effects of miR-124 on reversing resistance of CD133^+^ HCC cells to platinum-based chemotherapeutic drugs.** (**A**) Carboplatin IC50 to CD133^+^ and CD133^-^ HepG2 and Huh7 cells (*P<0.05). (**B**) Oxaliplatin IC50 to CD133^+^ and CD133^-^ HepG2 and Huh7 cells (*P<0.05). (**C**) Effects of miR-124 (50 pmol/mL) on decreasing carboplatin IC50 to CD133^+^ HepG2 and Huh7 cells (*P<0.05). (**D**) Effects of miR-124 (50 pmol/mL) on decreasing oxaliplatin IC50 to CD133^+^ HepG2 and Huh7 cells (*P<0.05).

The results of the cell viability assays showed that the CD133^+^ HepG2 and Huh7 cells exhibited resistance to carboplatin (Figure 8A) and oxaliplatin (Figure 8B) compared to the CD133^-^ HepG2 and Huh7 cells respectively. We then investigated the effects of miR-124 on carboplatin- and oxaliplatin-induced cytotoxicity against CD133^+^ HepG2 and Huh7 cells. We found that overexpression of miR-124 expression decreased the IC50 of CD133^+^ HepG2 and Huh7 cells to carboplatin (Figure 8C) and oxaliplatin (Figure 8D). Thus, we demonstrated that recovery of miR-124 expression can decrease the resistance of CD133^+^ HCC cells to platinum-based chemotherapy.

## DISCUSSION

In the treatment of HCC, platinum-based chemotherapy is commonly used in preoperative or postoperative chemotherapy. Sensitivity of chemotherapy severely determines the prognosis of HCC patients [32–34]. Cisplatin is an important member of platinum-based chemotherapeutic drug. In cancer cells, cisplatin induces DNA crosslink and thus inhibits DNA replication and induces DNA damage and cell apoptosis [35,36]. Therefore, cisplatin is commonly used in HCC treatment. However, drug resistance against cisplatin usually occurs in many cases [37,38]. It is urgent to explore the potential mechanisms and take strategies to reduce the cisplatin resistance in HCC.

MiRNAs have been reported to be dysregulated frequently in various cancers including HCC. Furthermore, miRNAs dysregulation has been found to be responsible for development of drug resistance in cancer cells [39,40]. Therefore, cancer-related miRNAs are potential targets in cancer therapy. MiR-124 has been reported to act as a tumor suppressor in some cancers. Cellular miR-124 can inhibit growth, invasion and tumorigenesis of gastric cancer, ovarian cancer, lung cancer and renal cell carcinoma. Downregulation of miR-124 usually predicts a poor prognosis for cancer patients [41–44]. Therefore, recovery of miR-124 may be a potential strategy in cancer therapy.

In the present study, we found that the population of CD133^+^ HCC cells showed significant resistance to cisplatin and some other platinum-based chemotherapeutic drugs compared to the CD133^-^ HCC cells. It suggested that the CD133^+^ HCC cells were responsible for drug resistance in HCC. After analysis of miR-124 expression between CD133^+^ and CD133^-^ HCC cells, we found the downregulation of miR-124 in CD133^+^ HCC cells. Next, we compulsively recovered the expression level of miR-124 in CD133^+^ HCC cells by transfection with miR-124 mimics. Interestingly, we found that co-treatment with miR-124 sensitized these CD133^+^ HCC cells to treatment of platinum-based chemotherapeutic drugs in vitro and in vivo. Our study demonstrated that overexpression of miR-124 may represent a novel strategy to overcome the drug resistance in CD133^+^ HCC cells.

SIRT1 is a histone deacetylase in cancer cells [45]. Overexpression of SIRT1 increases the cellular level of superoxide dismutase (SOD) which is a key cellular antioxidant, and thus eliminates the generation of ROS which is apoptosis promoter [29,46,47]. In the downstream pathway of ROS-dependent apoptosis, JNK is a key molecular linkage between oxidative stress and cell apoptosis. Activation of JNK induces the overexpression of pro-apoptotic proteins and inhibits the function of anti-apoptotic proteins [48,49]. Thus, SIRT1/ROS/JNK pathway is one of the important signals that regulates cell apoptosis in cancers.

In this study, we found that SIRT1 was overexpressed in CD133^+^ HCC cells. High level of SIRT1 prevented the generation of ROS induced by cisplatin treatment in HCC cells. Therefore, CD133^+^ HCC cells exhibited lower response to apoptosis compared to the CD133^-^ HCC cells. Furthermore, we found that the mechanism by which miR-124 sensitized the CD133^+^ HCC cells to cisplatin treatment was dependent on the decrease of SIRT1 expression. That is to say, SIRT1 was the target of miR-124 in HCC. Our results indicated that recovery of miR-124 decreased the SIRT1 expression and thus promoted the production of ROS and activation of JNK in the cisplatin-treated CD133^+^ HCC cells. And finally, overexpression of miR-124 resensitized the CD133^+^ HCC cells to cisplatin-induced apoptosis.

## CONCLUSION

This study indicated the effect of miR-124 on sensitizing cisplatin-induced cytotoxicity to CD133^+^ HCC cells in vitro and in vivo. The miR-124/SIRT1/ROS/JNK pathway may represent a potential target in attenuating the cisplatin resistance in CD133^+^ HCC cells.

## MATERIALS AND METHODS

### Cell culture

The HCC cell lines HepG2 and Huh7 were purchased from the Institute of Biochemistry and Cell Biology, Chinese Academy of Sciences (Shanghai, China) and maintained in RPMI-1640 (Gibco, USA) supplemented with 10% fetal bovine serum (FBS, Gibco) at 37°C in a humidified 5% CO_2_ incubator. For separation between CD133^+^ HCC cells and CD133^-^ HCC cells, HepG2 and Huh7 cells were incubated with fluorescein isothiocyanate (FITC) conjugated CD133 antibody (Miltenyi Biotec, Germany) for 20 min at room temperature. Subsequently, CD133^+^ and CD133^-^ HepG2 and Huh7 cells were sorted by FACS vantage (FACSCALIBUR, BD Biosciences, USA).

### Quantitative reverse transcriptase real time PCR (qRT-PCR)

Total RNAs of HCC cells were extracted with TRIzol reagent (Invitrogen, USA). For reverse transcription, One Step PrimeScript miRNA cDNA Synthesis Kit (Takara Bio, Inc., Otsu, Japan) was used to synthesize the cDNA of HCC cells according to the manufacturer’s instruction (Takara Bio, Inc., Otsu, Japan). qPCR was performed in triplicate by using the SYBR Premix Ex Taq II (Takara Bio, Inc.) on ABI PRISM 7900 Sequence Detection system (Applied Biosystems, USA). GAPDH gene was used as the internal reference to determine the relative expression of SIRT1, and the expression of miR-124 was determined according to the internal control of U6 snRNA. SIRT1 forward primer sequence: 5’-TGATGAACCGCTTGCT-3’, reverse sequence: 5’-TGGTCTTACTTTGAGGGA-3’. GAPDH forward primer sequence: 5’-ATCCCATCACCATCTTCC-3’, reverse sequence: 5’-GACCCTTTTGGCTCCC-3’.

### Transfection

Human miR-124 mimics (5’-UUAAGGCACGCGGUGAAUGC-3’), 2’-Omethyl modified miR-124 antisense oligonucleotide (anti-miR-124, 5’-GCAUUCACCGCGUGCCUUAA-3’) and negative control oligonucleotide (NCO, 5’-GUAGGAAUCGUGGCUACGCA-3’) were purchased from Genepharma Company (Shanghai, China). Open reading frame of SIRT1 gene was amplified by PCR and then inserted into the pcDNA3.1 (Invitrogen) to conduct the SIRT1 eukaryotic expression plasmid. SIRT1 siRNA (forward: 5’-GUAUUGCUGAACAGAUGGAUU-3’, reverse: 5’-UCCAUCUGUUCAGCAAUACUU-3’, Genepharma Company) and plasmid was used for loss-of-function and gain-of-function experiments, respectively. For transfection, miR-124 mimics (50 pmol/mL), anti-miR-124 (50 pmol/mL), NCO (50 pmol/mL), SIRT1 siRNA (50 pmol/mL) and SIRT1 plasmid (2 μg/mL) were transfected into the HCC cells by using Lipofectamine 2000 (Invitrogen) according to the instruction of the manufacturer.

### Cell viability and IC50

A total of 5 × 10^3^ transfected HCC cells were seeded in 96-well plates overnight for adherence. Then, the cells were treated with different concentrations of cisplatin for 48 h. After treatment, cells were incubated by 3-(4, 5-dimethylthiazol-2-yl)-2, 5-diphenyltetrazolium bromide (MTT) for 4 h. Subsequently, dimethyl sulfoxide (DMSO) was added and the absorbance in each well was measured at 570 nm using a microplate reader (Sunrise Microplate Reader, TECAN, Switzerland). Half maximal inhibitory concentration (IC50) of cisplatin was calculated according to the cell viability viability curves of HCC cells.

### Western blot analysis

Whole cell lysates were prepared by using RIPA buffer (Cell Signaling Technology, Beverly, USA). Equal amount of protein samples was separated by 10% sodium dodecyl sulfate polyacrylamide gel electrophoresis (SDS-PAGE) and then transferred to polyvinylidene ﬂuoride (PVDF) membranes (Millipore, Billerica, MA, USA). Subsequently, the membranes were probed by primary antibodies of SIRT1, GAPDH and phosphorylated JNK (p-JNK) (Cell Signaling Technology, USA) overnight. After incubation with appropriate secondary antibodies, the protein bands were detected by using an enhanced chemiluminescent substrate (Thermo Fisher Scientific, Inc, USA).

### Luciferase reporter assay

SIRT1 3’ UTR fragment sequence containing the predicted binding site paired with miR-124 was amplified and cloned into the downstream of firefly luciferase gene in the pMIR-REPORT™ miRNA Expression Reporter Vector (Thermo Fisher Scientific, Inc.) according to the manufacturer’s instruction. The recombinant plasmid was named as pMIR-wtSIRT1. To conduct the mutant SIRT1 3’ UTR-luciferase reporter plasmid, QuikChange Site-Directed Mutagenesis kit (Stratagene, USA) was used based on the pMIR-wtSIRT1 plasmid according to the manufacturer’s instruction. The recombinant plasmid was named as pMIR-mtSIRT1. To perform the luciferase reporter assays, cells were co-transfected with the pMIR plasmid, Renilla luciferase pRL-TK vectors (Promega, USA) and the miR-124 mimics by using the lipofectamine 2000. 48 h after incubation, luciferase activities were measured by using Dual-Luciferase Reporter assay system (Promega) according to the manufacturer’s instruction.

### Flow cytometry

Generation of reactive oxygen cluster (ROS) and cell apoptosis were measured by flow cytometry analysis. For detection of ROS, cells were stained with dihydroethidium (DHE) (Molecular Probes, USA) for 15 min at room temperature. After washing with PBS for three times, cells were analyzed by flow cytometry (Becton Dickinson, USA). For measurement of apoptosis, cells were incubated with the Annexin V/Propidium Iodide (PI) (Sigma-Aldrich, USA) for 15 min at room temperature according to the manufacturer's instructions. Subsequently, cells were collected and washed with PBS followed by analysis on flow cytometry.

### Tumor growth in nude mice

CD133^+^ HepG2 cells which were stably overexpressed miR-124 (LT-miR-124) were generated by using a lentiviral-based system through Genechem Co., Ltd and selectively cultured in 1 μg/mL puromycin for 2 weeks. CD133^+^ HepG2 cells which were transfected with empty lentivirus were used as control. For xenograft, 5×10^6^ transfected cells were injected subcutaneously into the right armpit of mice (four-week-old and female immunodefcient nude BALB/c mice (Shanghai Super-B&K Laboratory Animal Corp., Ltd., Shanghai, China). Cisplatin (5 mg/kg) were administrated by intraperitoneal injection twice a week when the xenografts reached 0.5 cm in diameter. The experimental animals were divided into four groups (8 mice/ group). The tumor volume (V) was calculated based on the following equation: 1/2 × length × width^2^. 28 days post inoculation, mice were sacrificed and the tumor tissues were separated. The animal care and experimental protocols were approved by the Animal Care Committee of Sun Yat-sen Memorial Hospital, Sun Yat-sen University.

### Statistical analysis

Data are represented as mean ± standard deviation (SD) and obtained from three independent experiments. Data were analyzed by using SPSS 15.0. For comparison analysis, two-tailed Student’s t-tests were used to estimate the statistical differences between two groups. One-way analysis of variance (ANOVA) and Bonferroni’s post hoc test were used to determine the differences between three or more groups. *P*<0.05 was considered to indicate a statistically significant difference.
